# Inflammation-associated microbiota in pediatric eosinophilic esophagitis

**DOI:** 10.1186/s40168-015-0085-6

**Published:** 2015-06-01

**Authors:** Alain J Benitez, Christian Hoffmann, Amanda B. Muir, Kara K. Dods, Jonathan M. Spergel, Frederic D. Bushman, Mei-Lun Wang

**Affiliations:** Division of Gastroenterology, Hepatology, and Nutrition, The Children’s Hospital of Philadelphia, Philadelphia, PA 19104 USA; Department of Microbiology, Perelman School of Medicine, University of Pennsylvania, 3610 Hamilton Walk, Philadelphia, PA 19104 USA; Division of Allergy and Immunology, The Children’s Hospital of Philadelphia, Philadelphia, PA USA; Department of Pediatrics, Perelman School of Medicine, University of Pennsylvania, Philadelphia, PA USA; Depto. de Alimentos e Nutrição Experimental, Faculdade de Ciências Farmacêuticas, Universidade de São Paulo, São Paulo, SP 05508-000 Brazil

## Abstract

**Background:**

Eosinophilic esophagitis (EoE) is an allergic disorder characterized by eosinophil-predominant esophageal inflammation, which can be ameliorated by food antigen restriction. Though recent studies suggest that changes in dietary composition may alter the distal gut microbiome, little is currently known about the impact of a restricted diet upon microbial communities of the oral and esophageal microenvironments in the context of EoE. We hypothesize that the oral and esophageal microbiomes of EoE patients are distinct from non-EoE controls, that these differences correspond to changes in esophageal inflammation, and that targeted therapeutic dietary intervention may influence community structure. Using 16S rRNA gene sequencing, we characterized the bacterial composition of the oral and esophageal microenvironments using oral swabs and esophageal biopsies from 35 non-EoE pediatric controls and compared this cohort to samples from 33 pediatric EoE subjects studied in a longitudinal fashion before and after defined dietary changes.

**Results:**

Firmicutes were more abundant in esophageal samples compared to oral. Proportions of bacterial communities were significantly different comparing all EoE esophageal microbiota to non-EoE controls, with enrichment of Proteobacteria, including *Neisseria* and *Corynebacterium* in the EoE cohort, and predominance of the Firmicutes in non-EoE control subjects. We detected a statistically significant difference between actively inflamed EoE biopsies and non-EoE controls. Overall, though targeted dietary intervention did not lead to significant differences in either oral or esophageal microbiota, reintroduction of highly allergenic foods led to enrichment in *Ganulicatella* and *Campylobacter* genera in the esophagus.

**Conclusions:**

In conclusion, the esophageal microbiome in EoE is distinct from that of non-EoE controls, with maximal differences observed during active allergic inflammation.

**Electronic supplementary material:**

The online version of this article (doi:10.1186/s40168-015-0085-6) contains supplementary material, which is available to authorized users.

## Background

Eosinophilic esophagitis (EoE) is a chronic Th2-mediated allergic disease of the esophagus characterized by eosinophilic esophageal infiltration. Its pathogenesis is incompletely understood and likely involves both environmental and genetic factors [1,2]. In 1995, Kelly *et al*. made the seminal observation that an amino acid based elemental diet led to complete resolution of EoE symptoms, esophageal eosinophilia, and associated endoscopic findings in pediatric patients [3]. Since this initial finding, other dietary treatment options for EoE have expanded to include targeted food elimination diets and the empiric six-food elimination diet (SFED), which has been shown to be an effective treatment for both children and adults [4-8]. Food antigens have since been identified as significant causative agents which trigger immune responses in EoE [9].

Here, we investigate the relationship of diet, the gut microbiome, and EoE. The effect of diet upon gut microbial composition has been explored previously in obesity [10-13] and inflammatory bowel disease (IBD) [10,14]. Relevant to EoE, in which a significant proportion of patients have IgE-mediated food allergies [2], Stefka *et al*. recently showed that the presence of specific gut commensals are protective against development of food allergy in mouse models [15], potentially through enhancing epithelial barrier function and expansion of FoxP3+ regulatory T cells (Tregs) [16].

Several human studies support the association between altered gut microbiota and the development of atopic disorders early in life [17-19]. However, the effects are not consistent and were temporary in several studies [20]. Early colonization with certain bacterial species may influence the maturation of secretory immunoglobulin A (IgA), which is associated with protection against development of food allergies [21]. A reduction in microbial diversity with increased proportions of *E. coli* and *C. difficile* may be associated with a higher risk of eczema and allergic sensitization [22]. Clinical studies have indicated that probiotics may have some limited positive effects on atopic dermatitis, supporting a role for the gut microbiome, but this has not been confirmed in other studies [20].

Until recently, the esophagus was considered to have few cultivatable bacterial species [23]. Initial descriptions of the esophageal microbiome were focused on comparisons between the oral and the esophageal microbiome [24,25]. Following the development of community profiling using sequence-based methods, others have shown that the normal human esophagus has a unique Gram positive (Type I) dominated bacterial signature, and that shifts to a Gram negative (Type II) esophageal microbiome may be linked to gastroesophageal reflux disease (GERD) and Barrett’s esophagus (BE) [26]. The bacterial profile of the normal esophagus was subsequently analyzed by Fillon *et al*. who used the esophageal string test (EST) to characterize the esophageal microbiome in children without esophageal pathology [27]. Using the EST, direct comparisons between the oral and esophageal microbiomes revealed significant genera differences between these microenvironments.

To date, there have been no reports characterizing the esophageal microbiome in EoE. Given the well-established causal relationship between dietary antigens and EoE disease activity, we hypothesized that the esophageal microbiome in EoE subjects would be distinct from subjects without EoE, and that there would be differences in the EoE microbiome following dietary manipulation, which influences disease activity. In this study, we characterize the esophageal microbiome in children with and without EoE using 16S rRNA gene sequencing. Because assessments of EoE disease activity are currently dependent upon endoscopic surveillance, we also compare the composition of the oral microbiome to the esophageal microbiome to determine whether the oral microbiome might serve as a surrogate for monitoring EoE disease activity.

## Results

### Patient characteristics

**Figure 1:**
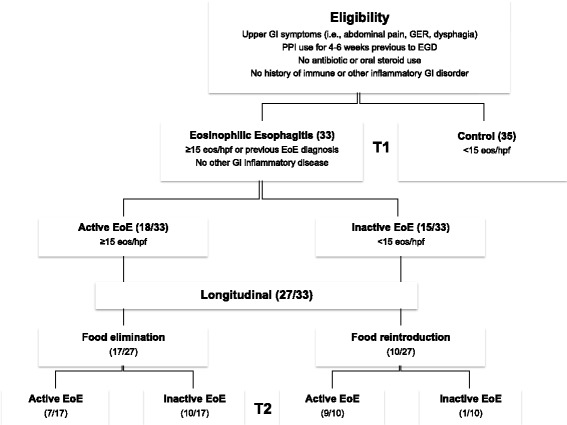
Flow chart of the experimental design.

Figure 1 summarizes the enrollment and sampling methods used in this study. Analysis was carried out for 68 subjects (59 males, 9 females) ranging from 2 to 18 years of age (Additional file 1: Table S1A and Additional file 2: Table S1B). All subjects had no history of antibiotic use for 4 to 6 weeks prior to the procedure, and the majority (66/68) of subjects were treated with a PPI for 4 to 6 weeks prior to the endoscopy. The two subjects who were not on PPI at the time of endoscopy were EoE patients who had been previously diagnosed with EoE while on appropriate PPI therapy.

Thirty-five subjects were designated as non-EoE controls. Within this group, 29/35 did not have any gastrointestinal pathology, and 6/35 had mild non-EoE esophagitis. In the EoE cohort (*N* = 33), 18 were classified as active EoE (≥15 eosinophils per hpf) and 15 were classified as inactive EoE (<15 eosinophils per hpf) at the first of two time-points. Among EoE subjects, 32/33 had no history of systemic or topical (swallowed) steroid use. Twenty-seven EoE subjects were enrolled in the longitudinal arm of the study. Seventeen EoE subjects with active disease at the first time-point underwent food elimination challenge (removal of a food(s) from the SFED), and 10 subjects with inactive disease at the first time-point added a food(s) into the diet from the SFED [7]. Food antigen removal or addition was continued for 4 to 8 weeks prior to the second time-point, at which point esophageal biopsies and oral swabs were obtained. In total, 88 oral samples and 78 esophageal samples were collected for analysis.

### The oral and esophageal microbiomes harbor distinct microbial populations

**Figure 2:**
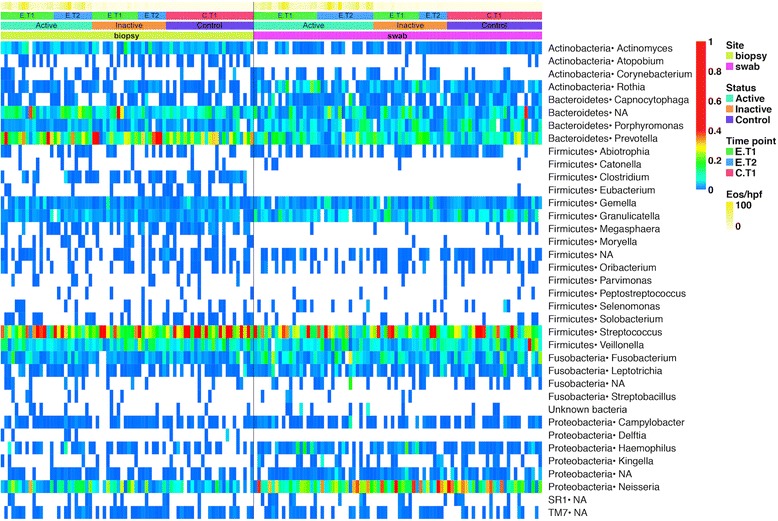
Heatmap of esophageal and oral microbiome. Each column corresponds to a specific sample, each row to a type of bacteria identified in the sequence data. The proportions that each lineage contributed to the full population within each sample are indicated with the color scale to the right of the figure (values from 0 to 1). Metadata is color-coded at the top, including site sampled, disease status, and number of eos/hpf (eosinophils per high power field) reported in the biopsies at the time of endoscopy. Time-points: Samples were obtained at single time-points in non-EoE controls (C.T1), and EoE subjects had two separate sample collections (first: E.T1, second: E.T2).

DNA was purified from biopsy or swab specimens, and the V1V2 region of the bacterial 16S rRNA gene was amplified by PCR. Products were sequenced using the 454/Roche method, then sequences condensed into operational taxonomic units of 97% sequence identity and nearest taxonomy assigned for each (Additional file 3: Table S2). Figure 2 shows a heatmap of samples, indicating the types of bacteria detected in each sample. Strong distinctions between esophageal and oral samples were observed. Specifically, several members of the phylum Firmicutes were detected almost exclusively in the biopsy samples, including *Clostridium*, *Eubacterium*, *Megasphaera*, *Mogibacterium*, and *Moryella*. In addition, the *Atopobium* genus of Actinobacteria was predominantly detected in esophageal biopsy samples and was absent in the majority of oral swabs.

Some members of the Bacteroidetes, Firmicutes, and Proteobacteria were detected in both sites regardless of phenotype including the genus *Prevotella*, *Streptococcus*, and *Neisseria*, respectively. We used a Mantel correlation and a Procrustes test to determine if there was a statistically significant correlation between the microbiome present in the two sites across patients. Both tests showed a significant correlation (Mantel correlation = 0.16, *P* value: 0.008; Procrustes R2: 0.15, *P* value: 0.009), indicating sharing of lineages between the oral and esophageal sites.

### The esophageal microbiome in active EoE is characterized by a distinct microbiome compared to non-EoE controls

We next investigated whether consistent patterns in community structure could be detected for EoE *versus* non-EoE controls within each sample type. We used UniFrac to calculate distances between all pairs of samples [28,29] then used tests of the distance matrices to assess relationships among communities from EoE and non-EoE control subjects. Communities were compared by membership (presence-absence information) using unweighted UniFrac and by relative abundance using normalized weighted UniFrac.

A permanova-based test showed a trend toward a difference between all EoE and non-EoE control esophageal samples in community proportions which did not reach statistical significance (weighted UniFrac distances; Adonis *P* values for EoE *versus* non-EoE control subjects: *P* = 0.06). This suggested a trend toward differences between sample groups resulting from differences in bacterial proportions (Additional file 4: Figure S1A,B,C)). Unweighted UniFrac revealed no differences in community membership (Adonis *P* values for EoE *versus* non-EoE control subjects: *P* = 0.52). Of note, no differences in membership were found in EoE subjects on a PPI (26/28) when compared to those not on a PPI (2/28). No significant differences were observed between EoE and non-EoE control groups in tests of the oral samples (data not shown).

To determine whether the differences between EoE and non-EoE microbial proportions were due to disease activity, we compared samples from individuals with active EoE, inactive EoE, and non-EoE controls. Using weighted UniFrac distances, there were statistically significant differences between active EoE and non-EoE control subjects. Differences between inactive EoE *versus* non-EoE controls in weighted UniFrac were not statistically significant (Adonis *P* values for active EoE *versus* non-EoE control subjects: 0.037; inactive EoE *versus* non-EoE control subjects: 0.062). Comparisons between active EoE and inactive EoE using weighted UniFrac did not show significant differences in either community membership or relative abundance (disease status Adonis *P* values: weighted UniFrac *P* = 0.404; unweighted UniFrac *P* = 0.171).

Comparisons of bacterial community membership using unweighted UniFrac showed no statistically significant differences (Adonis *P* values for active EoE *versus* non-EoE control: 0.124; inactive EoE × non-EoE control: 0.481). Together, this suggested that an active, eosinophil-rich, inflamed tissue is associated with a distinct shift in relative abundance of the esophageal microbiota, but not in community membership. Additionally, richness, Shannon diversity, and evenness indexes were calculated for each sample using a Wilcoxon rank-sum test (between non-EoE controls, active EoE, and inactive EoE subjects) and significant differences were not detected for any comparison (Additional file 3: Table S2 and Additional file 5: Figure S2).

Because a small number of esophageal samples in the non-EoE control cohort (six subjects) had histologic evidence of non-eosinophilic esophagitis, we performed a separate analysis to compare the six non-EoE control samples with esophagitis to the 25 non-EoE samples without esophageal inflammation. We did not detect a difference between subjects with and without inflammation (unweighted UniFrac *P* = 0.577 and weighted UniFrac *P* = 0.455). We performed an additional analysis comparing EoE to non-EoE controls after removing the six subjects with non-EoE esophageal inflammation from the control cohort, and found that though there was a slight reduction in overall significance [EoE *versus* non-EoE control (unweighted UniFrac *P* = 0.614 and weighted UniFrac *P* = 0.108), inactive EoE *versus* non-EoE control (unweighted UniFrac *P* = 0.53 and weighted UniFrac *P* = 0.137) and active EoE *versus* non-EoE control (unweighted UniFrac *P* = 0.186 and weighted UniFrac *P* = 0.065)], similar patterns in esophageal microbiota were observed. Based upon these findings, we continued to include the six subjects with non-EoE esophagitis as part of the non-EoE control cohort in the subsequent analyses.

### Bacterial lineages associated with EoE and non-EoE controls

**Figure 3:**
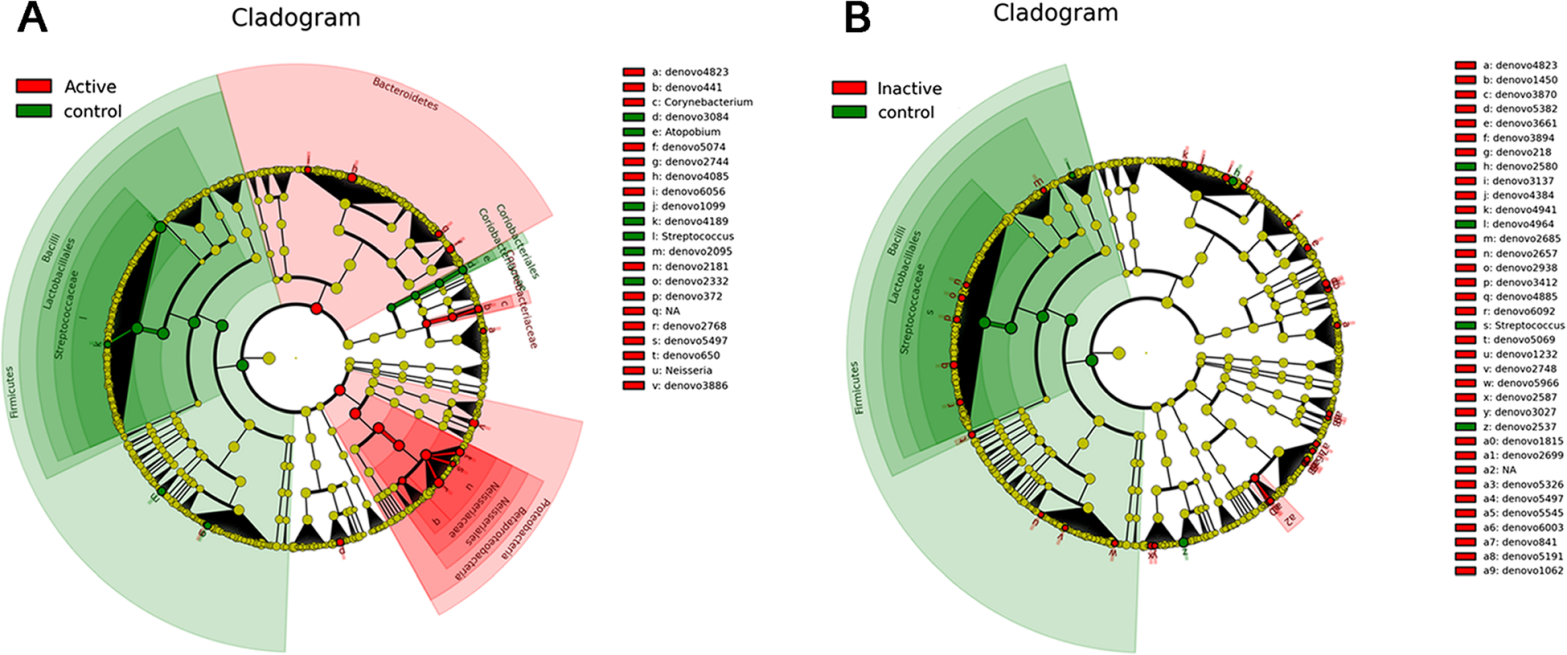
Comparison of lineages enriched in EoE (28) *versus* non-EoE control (31) esophageal samples using LEfSe. **(A)** Comparison of active EoE (21) *versus* non-EoE control (31). **(B)** Comparison of inactive EoE (20) *versus* non-EoE control (31). The “denovo” indication shows the OTU number.

**Figure 4:**
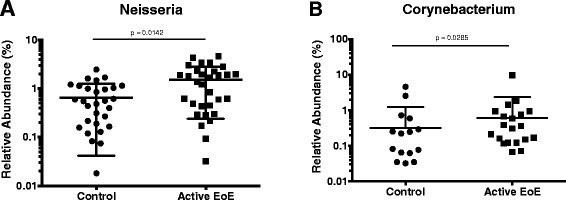
Relative abundance plots for *Neisseria* **(A)** and *Corynebacterium* **(B**) in active EoE samples compared to non-EoE controls.

We used the linear discriminant analysis effect size (LEfSe) test to determine which taxonomic groups were responsible for the changes observed with weighted UniFrac in EoE [30]. Taxonomic cladograms of OTUs comparing active EoE (21 samples) *versus* non-EoE control (31 samples) and inactive EoE (20 samples) *versus* non-EoE control (31 samples) are shown in Figure 3. The *Neisseria* genus of the Proteobacteria was enriched in the EoE samples, as was *Corynebacterium*. A relative abundance plot showing the major genera enriched in active EoE samples compared to non-EoE controls is shown in Figure 4. These lineages have been previously implicated in inflammation at other body sites [31,32], providing a parallel with EoE. The *Streptococcus* and *Atopobium* genera were consistently enriched in non-EoE control samples. Relative abundance plots for lineages enriched in non-EoE controls compared to active EoE are shown in Additional file 6: Figure S3.

### Effect of dietary elimination on the esophageal microbiome in EoE

The SFED is an established treatment for EoE, with outcomes that compare favorably with both targeted elimination diets and an elemental diet. Following the establishment of a SFED, an EGD is typically performed to establish reduction in esophageal eosinophilia. Once endoscopic remission is established, groups of foods from the SFED are added back into the diet and surveillance EGD is performed several weeks after addition of the new food group. In our patient cohort, we included subjects before (time-point 1) and after (time-point 2) a clinically recommended diet change and hypothesized that dietary manipulation using foods from the SFED would not only lead to alterations in the esophageal inflammatory state but would also change esophageal microbial composition.

**Figure 5:**
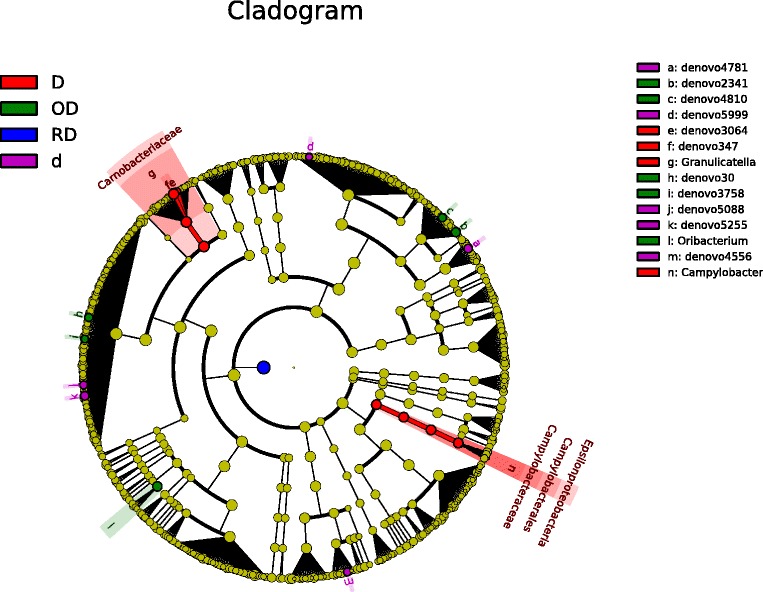
Comparison of lineages enriched under different diet regimens. D = addition of food from the six-food elimination diet (SFED) (10); OD = open diet (4); RD = restricted diet (13); d = removal of food from the SFED (7).

**Figure 6:**
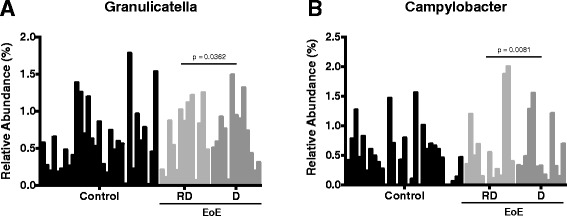
Relative abundance for *Granulicatella* **(A)** and *Campylobacter* **(B)** in non-EoE controls and subjects before (RD) and after (D) addition of food from the SFED.

A permanova test, using the weighted and unweighted UniFrac distances, was used to determine if dietary interventions could change the disease status and esophageal microbiome over time,. Dietary intervention did not have an appreciable global effect on the UniFrac distances (dietary intervention Adonis *P* values: weighted UniFrac *P* = 0.220; unweighted UniFrac *P* = 0.450), although this could be explained by the small effective number of patients used in this specific analysis. Follow up analysis using LEfSe was carried out to determine if specific lineages were differentially represented following specific dietary changes in EoE [33]. Following the addition of foods from the SFED, there was an enrichment in two OTUs of the *Granulicatella* genus, Carnobacteriaceae family and the *Campylobacter* genus in the esophageal microenvironment (Figure 5) (Granulicatella.denovo347: *P* < 0.0363; Granulicatella.denovo3064: *P* < 0.0358; *Granulicatella*: *P* < 0.0362; *Campylobacter*: *P* < 0.0081. Raw Kruskal-Wallis *P* values from significant features detected using LEfSe). Corresponding relative abundance plots for *Granulicatella* and *Campylobacter* in subjects with a restricted diet who later added an allergenic food as compared to non-EoE controls are shown in Figure 6.

## Discussion

In this study, we report a characteristic esophageal microbiome in pediatric EoE patients that is influenced by EoE disease activity and is distinct from the esophageal microbiome of non-EoE control subjects. Diet changes did not detectably influence the composition of the esophageal microbiome, but the sample was small and heterogeneous, so further work is needed in this area. We also show a modest but significant correlation between the oral and esophageal microbiomes in the cohort used in this study, regardless of disease status.

The esophageal microbiome of non-EoE control subjects showed a predominance of Gram (+) bacteria of the *Streptococcus* genus. This is in agreement with previous findings in control adult subjects [33] and pediatric subjects [27] without esophageal inflammation. Subjects included in the study by Yang *et al*. were predominantly elderly males [33], whereas the majority of subjects in the analysis by Fillon *et al*. were pediatric females [27], showing consistency between age groups and genders. In contrast to both studies, the majority of our subjects are pediatric males with nearly 100% concurrent and documented PPI use, providing a further indication of the resilience of the *Streptococcus*-dominated microbiome in the uninflamed esophagus in all age groups and genders.

We observed differences in the esophageal microbiota when comparing samples from active EoE to non-EoE control subjects. Similar to previously published findings, members of the genera *Corynebacterium* and *Neisseria* of the phylum Proteobacteria were enriched in the setting of esophageal inflammation [24,34,35]. The presence of Gram (-) organisms including *Neisseria* has been reported in previous studies of the esophagus [26,36] and correlated to inflammatory states. However, in contrast to these studies which characterized adult non-EoE GERD cohorts, ours is the first to explore microbial communities specifically in EoE. While our results might suggest a common microbiome shared by all forms of esophageal inflammation, our study was limited by a small number of subjects with non-EoE esophageal inflammation making it challenging to address if the presence of non-EoE related inflammation could affect such correlations. Interestingly, however, the microbial composition of our very small cohort of non-EoE controls with esophageal inflammation was not distinct from those without esophageal inflammation and was still distinct from microbiota present in inflamed mucosa of subjects with EoE. A future larger study specifically comparing the EoE to non-EoE (GERD) esophageal microbiome would be highly informative in this regard.

Understanding the cause and effect relationship between disease state and the microbiome is a subject of great debate addressed in gnotobiotic studies. Baumler and colleagues found that *Salmonella* is increased in gut inflammation and proposed that inflammation promotes proliferation of *Salmonella* by production of compounds that *Salmonella* can use as terminal electron acceptors [37,38]. In different disease states, gnotobiotic mouse models have been used to determine whether gut commensals provoke or prevent inflammation. While enteric bacteria are necessary for the development of murine colitis [39], germ-free mice are more prone to the development of allergen sensitization [40,41]. EoE poses a unique challenge in this area, as it is clinically characterized by food allergen sensitization and chronic inflammation.

Although we were unable to detect global differences between subjects on an unrestricted diet and those that added or removed a highly allergenic food, our LEfSe analysis revealed a possible enrichment of two genera, *Campylobacter* and *Granulicatella,* following the addition of a food antigen in the SFED. *Campylobacter* species have been associated with inflammatory states of gastrointestinal tract including periodontal disease, Barrett’s esophagus, and IBD [34]. This data suggests addition of foods leads to increased inflammation and changes in the microbiome. Others found that seropositivity to *Campylobacter jejuni* had a greater association to the development of atopy [42]. The specific correlation of these species in the pathogenesis of EoE requires further study.

The dietary additions and eliminations in this study were performed according to the clinical recommendations of the primary gastroenterology/allergy team and foods which were added or eliminated varied among EoE subjects. Although an initial goal of our study was to compare the microbiome of EoE subjects before and after an amino acid based elemental diet, the number of patients placed on an elemental diet at our institution and others has declined significantly due to the recently proven efficacy of the SFED [5,8,43]. Therefore, we did not have adequate power to determine the effects of specific foods upon the differences in the microbiome. The effect of milk will be fascinating to study in the future as it is the most common allergen in EoE [8] and has a definite effect on gut microbiota [44].

Previous studies have characterized the oral microbiome in the absence of inflammation. The oral microbiomes of children and adults show a predominance of Firmicutes, Proteobacteria, Actinobacteria, and Fusobacteria [45]. In other studies, *Streptococcus*, *Prevotella*, *Neisseria*, *Haemophilus*, *Porphyromonas*, *Gemella*, *Rothia*, *Granulicatella*, *Fusobacterium*, *Actinomyces*, *Veillonella*, *and Aggregatibacter* are predominant organisms in saliva of healthy subjects [46]. Comparisons between the oral and esophageal flora have been previously reported, showing predominance of *Streptococcus*, *Fusobacterium*, *Neisseria*, *Haemophilus*, and *Prevotella* in both sites [25]. This is consistent with our results in which *Streptococcus, Neisseria*, and *Prevotella* were predominant organisms in both the oral and esophageal environments.

In the current study, we aimed to study the correlation between these two microbial populations and determine whether the 16S rRNA gene tag analysis of the oral mucosa could serve as a biomarker of disease activity. By having a single sample per individual, we are able to avoid autocorrelation issues arising from having two samples from the same individual. The Mantel correlation and Procrustes values suggest a weak, albeit significant, correlation between the two datasets (oral and esophageal biota). We did not detect differences in the oral microbiome between active EoE and inactive EoE or non-EoE control samples, suggesting that in pediatric EoE, bacterial communities are stable and might not be altered by dietary modification. Thus, the data do not support use of oral samples in lieu of biopsies for EoE surveillance.

## Conclusions

We report distinctive microbiota in patients with active EoE compared to non-EoE controls. No significant differences were seen in inactive EoE samples compared to non-EoE controls. This difference suggests that the increase in *Neisseria* and *Corynebacterium* may be due to inflammation and not EoE itself. Our subjects were treated with a variety of dietary interventions. Although this study is underpowered to detected differences among the treatments, some possible effects were observed that can serve as hypotheses for future studies.

## Methods

### Subjects

Subjects enrolled in the study (IRB# 10-007737) were undergoing diagnostic esophagogastroduodenoscopy (EGD) at The Children’s Hospital of Philadelphia (CHOP). Inclusion criteria included an age between 6 months and 21 years, ongoing proton pump inhibitor (PPI) use for at least 4 weeks prior to EGD, no antibiotic use for at least 4 weeks prior to EGD, and no other esophageal disease or chronic inflammatory diseases of the gastrointestinal (GI) tract. Non-EoE control subjects showed no histopathologic abnormalities in the esophagus or distal GI tract and were not previously diagnosed with EoE. Both newly diagnosed EoE subjects and subjects who had previously been diagnosed with EoE based on clinical guidelines [2] were recruited and analyzed at two different time-points, before and after a specific diet change based upon recommendations of the gastroenterologist and/or allergist. At the first time-point, subjects were either on an open diet without restrictions (OD) or a restricted diet (RD) in which specific food antigens had already been removed based upon suspected causality in the subject’s EoE disease activity. Prior to the second time-point, selected foods from the SFED were either added to the diet (D) or eliminated from the diet (d). The experimental plan is shown in Figure 1, and subject metadata are shown in Additional file 1: Tables S1A and Additional file 2: Table S1B.

All EoE subjects were further stratified as active (≥15 eosinophils/high-power field (hpf)) or inactive (<15 eosinophils/hpf) based on EoE diagnostic criteria [2]. After written informed consent and/or assent was obtained, oral swabs from the inner cheeks, hard palate, and distal third of the tongue were collected using a sterile microbrush and stored on dry ice prior to processing. Esophageal biopsies from the distal third of the esophagus were collected during EGD using sterile forceps and immediately placed on dry ice.

### Sample processing

Oral swabs and esophageal biopsies were separately stored in sterile Eppendorf tubes on dry ice, then transferred to a −80°C freezer until processing. Bacterial DNA extraction from oral swabs and esophageal biopsies was performed in a sterile tissue culture hood using the MoBio PowerSoil DNA Isolation Kit (Mo Bio Laboratories, Carlsbad, CA, USA) and DNeasy Blood and Tissue Kit (Qiagen, Maryland, USA), respectively.

For bacterial DNA isolation from oral swabs, sterile scissors were used to cut the swab tip into the MoBio Power-Bead tube, and DNA was isolated according to manufacturer’s instructions. For extraction of bacterial DNA from biopsies, samples were incubated in a lysozyme solution containing 20 mg/ml lysozyme in 180 μl of 20 mM Tris-HCl (pH 8.0), 2 mM EDTA, and 1.2% Triton X-100, at 95°C for 5 min and 37°C for 60 min, followed by manufacturer’s instructions to complete DNA extraction. DNA was amplified by adding 10 μl of DNA to 40 μl of PCR mixture containing 5 μl of 10× PCR Buffer (Invitrogen, Carlsbad, CA, USA), 200 μM each dNTP, 50 pmol barcoded primer [47], and 2 units of AccuPrime TaqDNA polymerase. Reactions were performed on an Eppendorf Mastercycler pro Model 6325 (Hauppauge, NY, USA) using the following conditions: initial denaturation at 95°C for 5 min followed by 30 cycles (30 for swabs and 35 for biopsies) of 95°C × 30 s, 56°C × 30 s, and 72°C × 1 min 30 s. The reaction was terminated after an 8-min extension at 72°C. The amplification reactions were performed in quadruplicate. Products were pooled and bead purified using magnetic beads from Angencourt AMPure XP (Beckman Coulter Inc., Brea, CA, USA) and a DynaMag-Spin Magnetic Particle Concentrator (Invitrogen Dyna AS, Oslo, Norway).

### DNA sequencing and bioinformatics analysis

Pooled DNA samples were sequenced using the Roche/454 Genome sequencer or Genome Sequencer Junior platforms (454, Branford, CT, USA). The sequences obtained were processed using the QIIME software package [48] using default parameters and the R statistical package [49] unless otherwise stated. Briefly, sequences were collapsed into operational taxonomic units (OTUs) at 97% similarity, from which a representative sequence was selected. These representative sequences were used for taxonomic classification, OTU table creation, and UniFrac calculations. Samples yielding less than 300 sequences and OTUs containing only one sequence across all samples were removed from further analysis [50]. A multiple rarefaction procedure was implemented as follows to increase the confidence that the community assessed was a representative of the actual community present in the sample and to remove biases caused by uneven sampling depth across all samples: after filtering, the remaining samples were rarefied down to 500 sequences 100 times, and an average of the sequence counts for these rarefactions was used. The final OTU table was filtered to contain OTUs which were present in at least ten samples (Additional file 3: Table S2).

### Statistical analysis

Global microbiome changes were analyzed using weighted and unweighted UniFrac distances across all samples. Statistical differences were assessed using the Adonis test implemented in the R package Vegan v2.0-10 [51]. Adonis permutations were restricted using strata to account for the repeated measure nature of the data when testing the disease status and dietary intervention variables. Richness and evenness were also assessed using the Vegan Package. For the dietary intervention test, only samples within diets corresponding to open diet (*N* = 4), restricted diet (*N* = 13), elimination (*N* = 7), and reintroduction of an allergenic food (*N* = 10) were considered in order to reduce variability in the dataset. OTU differences across groups were analyzed using LEfSe [30]. Only one sample per subject per site of sampling was used in the statistical tests unless otherwise stated.

## Additional files

Additional file 1: Table S1A. Characteristics of non-EoE control pediatric subjects. Data include site of sample collection, disease status, symptoms, demographics, history of atopic disease, diet, endoscopic findings, histologic findings, and peak eosinophils per high power field. Additional file 2: Table S1B. Characteristics of EoE pediatric subjects. Data includes site of sample collection, time-point of data collection, disease status (active ≥ 15 eosinophils/high power field (hpf)) or inactive (<15 eosinophils/hpf), symptoms, demographics, history of atopic disease, specific dietary interventions, duration of intervention, endoscopic findings, histologic findings, and peak eosinophils per hpf. Additional file 3: Table S2. OTU table with taxa, sequence numbers per samples, Shannon diversity, and evenness values. Bacterial DNA sequences were condensed into OTUs of 97% sequence identity, and nearest taxa were assigned to each sequence. OTUs listed were present in at least ten samples. Additional file 4: Figure S1. Ordination comparing EoE and non-EoE control samples. (A) Pairwise distances were calculated between samples, then data plotted using Principal Coordinates Analysis. The centroid of the non-EoE control samples and the EoE samples are indicated on the plot. Lines connect each sample to the appropriate centroid. (B) and (C) show the same plot color-coded according the proportion of *Neisseria* and *Streptococcus* present in each sample. Additional file 5: Figure S2. Richness, Shannon diversity, and evenness indexes. Comparisons between control, inactive EoE, and active EoE using a Wilcoxon rank-sum test. Additional file 6: Figure S3. Relative abundance for genus *Streptococcus* and *Atopobium* in active EoE *versus* non-EoE controls.

## Abbreviations

EGD: Esophagogastroduodenoscopy
EoE: Eosinophilic Esophagitis
EST: Esophageal string test
GERD: Gastroesophageal reflux disease
HPF: High-power field
IBD: Inflammatory Bowel Disease
IgA: Immunoglobulin A
LEfSe: linear discriminant analysis effect size
PPI: Proton pump in inhibitor
SFED: Six-food elimination diet
Tregs: regulatory T cells
