# Supplementary material for: Inflammation-associated microbiota in pediatric eosinophilic esophagitis
Source: Microbiome. 2015 Jun 1;3:23. doi: 10.1186/s40168-015-0085-6 (PMC4450515; doi:10.1186/s40168-015-0085-6)
Supplement: Additional file 1: Table S1A. — Characteristics of non-EoE control pediatric subjects. Data include site of sample collection, disease status, symptoms, demographics, history of atopic disease, diet, endoscopic findings, histologic findings, and peak eosinophils per high power field. [file 40168_2015_85_MOESM1_ESM.pdf]

Non-EoE Control													
**ID**	**Site**	**Status**	**Symptoms**	**Age**	**Gender**	**Race**	**Atopy**	**FA**	**Steroids**	**Diet**	**EGD Findings**	**Histologic Findings**	**Eos**
**C01**	O	Control	GER	10y 7m	M	C	none	N	N	Open diet	Normal	No pathologic diagnosis	0
**C02**	OE	Control	none	2y 1m	F	C	unknown	N	N	Open diet	Normal	No pathologic diagnosis	0
**C03**	OE	Control	GER	5y 11m	M	C	unknown	N	N	Open diet	Normal	No pathologic diagnosis	0
**C04**	OE	Control	GER, abdominal pain	6y 6m	M	C	Asthma	N	N	Open diet	Normal	No pathologic diagnosis	0
**C05**	OE	GERD	abdominal pain, GER	15y 3m	F	C	none	N	N	Open diet	Normal	Squamous mucosa showing rare intraepithelial eosinophils	2
**C06**	OE	Control	GER	16y 1m	M	C	none	N	N	Open diet	Normal	No pathologic diagnosis	0
**C07**	OE	Control	none	17y 2m	M	C	Asthma	N	N	Open diet	Furrows	No pathologic diagnosis	0
**C08**	O	Control	abdominal pain	5y 4m	F	C	none	N	N	Open diet	Normal	No pathologic diagnosis	0
**C09**	OE	GERD	obstructive, GER	9y 2m	M	C	AD	N	N	Open diet	Normal	Squamous mucosa with a focus of infiltrating lymphocytes and single eosinophils compatible with mild esophagitis	1
**C10**	OE	Control	obstructive, GER	10y 11m	M	C	none	N	N	Open diet	Normal	No pathologic diagnosis	0
**C11**	OE	Control	abdominal pain, GER	8y 7m	M	AA	AD, Asthma	N	N	Open diet	Normal	No pathologic diagnosis	0
**C12**	O	Control	abdominal pain	7y 3m	M	C	none	N	N	Open diet	Normal	No pathologic diagnosis	0
**C13**	OE	Control	abdominal pain, GER	7y 4m	M	C	none	N	N	Open diet	Normal	No pathologic diagnosis	0
**C14**	OE	Control	abdominal pain, GER	11y 3m	M	C	Asthma	N	N	Open diet	Normal	No pathologic diagnosis	0
**C15**	OE	GERD	GER	13y 3m	M	C	AD, AR, Asthma	N	N	Open diet	Normal	Reactive squamous mucosa with few intraepithelial eosinophils consistent with mild esophagitis	3
**C16**	E	Control	abdominal pain, GER	7y 2m	M	C	Asthma	N	N	Open diet	Normal	No pathologic diagnosis	0
**C17**	E	Control	abdominal pain, GER	6y 8m	M	C	none	N	N	Open diet	Normal	No pathologic diagnosis	0
**C18**	OE	Control	none	4y 8m	M	C	Asthma	N	N	Open diet	Normal	No pathologic diagnosis	0
**C19**	OE	Control	none	14y 5m	M	C	Asthma	N	N	Open diet	Normal	No pathologic diagnosis	0
**C20**	OE	Control	obstructive	17y 6m	M	C	none	N	N	Open diet	Furrows	No pathologic diagnosis	0
**C21**	OE	Control	obstructive, GER	16y 11m	M	AA	AD	N	N	Open diet	Normal	No pathologic diagnosis	0
**C22**	OE	Control	abdominal pain	16y 4m	M	C	none	N	N	Open diet	Normal	No pathologic diagnosis	0
**C23**	OE	Control	none	14y 8m	M	C	Asthma	N	N	Open diet	Normal	No pathologic diagnosis	0
**C24**	OE	Control	abdominal pain, GER	4y 0m	M	AA	AD, Asthma	N	N	Open diet	Normal	No pathologic diagnosis	0
**C25**	OE	Control	GER	9y 5m	M	C	Asthma	N	N	Open diet	Normal	No pathologic diagnosis	0
**C26**	OE	Control	GER	4y 4m	M	C	AD	N	N	Open diet	Normal	No pathologic diagnosis	0
**C27**	OE	Control	obstructive, GER	10y 6m	M	C	AD	N	N	Open diet	Normal	No pathologic diagnosis	0
**C28**	OE	GERD	GER	10y 0m	M	C	Asthma	N	N	Open diet	Normal	Mild to moderate esophagitis with increased intraepithelial lymphocytes and a rare eosinophil	1
**C29**	OE	GERD	abdominal pain	9y 5m	M	C	none	N	N	Open diet	Normal	Squamous mucosa with rare single infiltrating eosinophils and a few lymphocytes, mild esophagitis	1

**C30**	OE	Control	GER	9y 11m	M	C	none	N	N	Open diet	Normal	No pathologic diagnosis	0
**C31**	OE	Control	none	6y 4m	M	C	none	N	N	Open diet	Normal	No pathologic diagnosis	0
**C32**	OE	Control	abdominal pain	11y 2m	F	C	none	N	N	Open diet	Normal	No pathologic diagnosis	0
**C33**	O	Control	none	2y 9m	M	C	Asthma	N	N	Open diet	Normal	No pathologic diagnosis	0
**C34**	OE	Control	abdominal pain, GER	8y 5m	M	C	AD, Asthma	N	N	Open diet	Normal	No pathologic diagnosis	0
**C35**	OE	GERD	obstructive	8y 7m	M	C	AD	N	N	Open diet	Normal	Squamous mucosa with a single intraepithelial eosinophil	1

A: Active
I: Inactive
AA: African American
AD: atopic dermatitis
AR: allergic rhinitis
C: Caucasian
E: esophageal
EGD: esophagoduodenoscopy findings
Eos: eosinophils per high power field
FA: food allergies (by skin prick or patch testing)
GER: gastroesophageal reflux
GERD: gastroesophageal reflux with esophagitis
O: Oral
OE: oral and esophageal
