# Supplementary material for: Inflammation-associated microbiota in pediatric eosinophilic esophagitis
Source: Microbiome. 2015 Jun 1;3:23. doi: 10.1186/s40168-015-0085-6 (PMC4450515; doi:10.1186/s40168-015-0085-6)
Supplement: Additional file 2: Table S1B. — Characteristics of EoE pediatric subjects. Data includes site of sample collection, time-point of data collection, disease status (active ≥ 15 eosinophils/high power field (hpf)) or inactive (<15 eosinophils/hpf), symptoms, demographics, history of atopic disease, specific dietary interventions, duration of intervention, endoscopic findings, histologic findings, and peak eosinophils per hpf. [file 40168_2015_85_MOESM2_ESM.pdf]

Eosinophilic Esophagitis															
**ID**	**Point**	**Site**	**Status**	**Symptoms**	**Age**	**Gender**	**Race**	**Atopy**	**FA**	**Steroids**	**Dietary Intervention**	**Duration (months)**	**EGD findings**	**Histologic Findings**	**Eos**
**E01**	**1**	O	I	none	4y 6m	M	C	Asthma	Y	N	restricting milk and oat	34	Normal	Mild esophagitis with few infiltrating lymphocytes and eosinophils	3
	**2**	O	A	none	7y 3m	M	C			N	added milk		Furrows	Severe esophagitis characterized by innumerable intraepithelial eosinophils associated with marked reactive epithelial changes	100
**E02**	**1**	OE	I	unknown	6y 9m	M	C	AR	Y	N	added oat and restricting milk, egg, peanut, beef, chicken, pork, apple, cinnamon, pecans, fish, shellfish, lamb, soy	4	Normal	No pathologic diagnosis	0
	**2**	OE	A	none	7y 1m	M	C			N	added soy		Furrows	Numerous intraepithelial eosinophils and severe reactive epithelial changes	50
**E03**	**1**	OE	I	none	9y 0m	M	C	AD, AR, Asthma	Y	N	restricting milk, egg, shellfish, chicken, spinach, peas, citrus, turkey, kiwi, pineapple, soy, coconut, corn, rice, blueberry, apple, pear, barley, wheat, lamb, beef, pumpkin, pork, green beans	3	Normal	Very mild esophagitis with few infiltrating eosinophils	2
	**2**	OE	A	unknown	9y 3m	M	C			N	added corn		Esophagitis	Moderately inflamed squamous mucosa	20
**E04**	**1**	OE	I	none	5y 7m	M	Other	AR, Asthma	Y	N	added corn and tomato and restricting peanuts, egg, tree nuts, sesame seeds, soy, wheat, beef, chicken, pork, turkey, potato, carrot, green beans, milk, rice, oats, peach, shellfish	Single time point	Normal	No pathologic diagnosis	0
**E05**	**1**	OE	I	none	2y 0m	F	C	unknown	Y	N	restricting milk, egg, wheat, soy, rice, barley, meats, peanuts, oat, white potato, corn	Single time point	Normal	Rare intraepithelial eosinophils	2
**E06**	**1**	E	I	none	17y 9m	M	AA	AR	Y	N	restricting milk, beef, peanut, tree nuts, shellfish	4	Normal	No pathologic diagnosis	0
	**2**	E	I	unknown	18y 0m	M	AA			N	added milk			No pathologic diagnosis	0
**E07**	**1**	OE	A	obstructive	15y 5m	F	C	AR, Asthma	Y	N	restricting milk and wheat	3	Furrows, rings, thickened folds	Esophagitis with reactive changes and variable intraepithelial eosinophils	15
	**2**	OE	I	unknown	15y 9m	F	C			Y	added milk and wheat		Furrows	No pathologic diagnosis	0

**E08**	**1**	OE	A	obstructive, GER	15y 2m	M	C	Asthma	N	N	open diet	3	Furrows, rings	Esophagitis with reactive squamous mucosa and intraepithelial eosinophils	18
	**2**	OE	I	unknown	15y 5m	M	C			N	removed oat, peanuts and soy		Furrows	Mild esophagitis with rare intraepithelial eosinophils and lymphocytes associated with reactive squamous mucosa.	3
**E09**	**1**	OE	A	GER	4y 4m	F	C	AD	Y	N	added corn and restricting egg, milk, wheat, soy, peanut, beef, chicken, turkey, salmon, garlic, potato	Single time point	Erythema, furrows	Moderate to severe esophagitis with superficially clustered eosinophils and reactive epithelial changes.	20
**E10**	**1**	OE	I	GER	9y 3m	M	C	AR, Asthma	Y	N	added corn and restricting peanut, egg, peas, carrot, sweet potato, tree nuts, milk	12	Normal	Mild esophagitis with eosinophils and reactive epithelial changes	6
	**2**	O	A	none	10y 2m	M	C			N	added milk		Furrows	Esophagitis with intraepithelial eosinophils, lymphocytes and reactive epithelial changes	25
**E11**	**1**	OE	A	obstructive, GER	12y 0m	M	C	none	Y	N	added egg and restricting peanuts	3	Furrows	Lymphocytic and eosinophilic infiltrate with little reactive epithelial changes	28
	**2**	OE	I	unknown	12y 3m	M	C			N	removed egg			Minimally inflamed squamous mucosa	3
**E12**	**1**	O	A	obstructive	10y 4m	M	C	AD, Asthma	Y	N	restricting soy, peanut, tree nuts	2	Furrows, rings, thickened folds	Intraepithelial eosinophils and marked reactive epithelial changes	18
	**2**	O	A	unknown	10y 6m	M	C			Y	removed milk		Edema, furrows, rings	Moderate esophagitis with variable infiltrating lymphocytes and eosinophils and reactive epithelial changes	60
**E13**	**1**	OE	I	obstructive	18y 8m	M	C	AR, Asthma	Y	N	Restricting all food and added amino acid based elemental formula	2	Edema, furrows	Few infiltrating eosinophils and mild reactive epithelial cells	6
	**2**	O	A	unknown	18y 10m	M	C			N	added celery, cucumber, lettuce and beef		Normal	Esophagitis with variable numbers of intraepithelial eosinophils	50
**E14**	**1**	OE	A	GER	18y 8m	M	C	AR, Asthma	Y	Y	removed milk and restricting soy, pork, egg, tree nuts	3	Normal	Focally increased intraepithelial eosinophils	17
	**2**	OE	I	unknown	18y 11m	M	C			N	added egg		Normal	Minimally inflamed squamous mucosa with scattered intraepithelial eosinophils	0
**E15**	**1**	E	I	none	6y 5m	M	C	AD, AR, Asthma	Y	N	removed corn and carrots and restricting milk, eggs, tree nuts, pork, wheat, beef, corn, soy, barley, green beans, pear, banana, sweet potato, peanut, coconut, carrot	Single time point	Normal	Squamous mucosa with some intraepithelial eosinophils	12

**E16**	**1**	OE	A	obstructive, abdominal pain	17y 3m	M	C	AD, AR, Asthma	Y	N	restricting peanuts and turkey	4	Furrows	Inflamed squamous mucosa with intraepithelial eosinophils and reactive epithelial cells	15
	**2**	E	I	none	17y 7m	M	C			N	open diet		Furrows	No pathologic diagnosis	0
**E17**	**1**	OE	I	none	3y 2m	M	AA	AD, Asthma	Y	N	added turkey, white potato, sweet potato, oat, carrot, broccoli, grapes, bananas, string beans, tomato, apple, honey, cinnamon and also restricting milk, peanuts and tree nuts	Single time point	Normal	Minimally inflamed squamous mucosa	1
**E18**	**1**	OE	I	abdominal pain	7y 10m	M	C	AD, Asthma	Y	N	restricting wheat, egg, pork and milk	2	Normal	Mildly inflamed squamous mucosa	3
	**2**	OE	A	none	8y 0m	M	C			N	added wheat		Normal	Mild to moderate esophagitis and reactive epithelial changes	15
**E19**	**1**	OE	I	none	7y 6m	M	C	none	Y	N	added pork and restricting milk, turkey, beef, sunflower oil	4	Normal	Squamous mucosa with rare intraepithelial eosinophils	3
	**2**	OE	A	none	7y 10m	M	C			N	added beef		Furrows, plaques	Reactive squamous mucosa with intraepithelial eosinophils with microabscess formation consistent with esophagitis	100
**E20**	**1**	OE	I	obstructive	9y 1m	M	C	AD, AR, Asthma	Y	N	added white potato and restricting egg, milk, wheat, soy, beef, corn, chicken, garlic, green beans, tree nuts, peanuts, peas, sesame seeds, tomato, apples, rice, tuna, turkey	5	Furrows	Moderate to severe esophagitis with variable eosinophil infiltrate and reactive epithelial changes.	12
	**2**	OE	A	other	9y 7m	M	C			N	added corn		Furrows	Esophagitis associated with reactive epithelial changes	60
**E21**	**1**	OE	I	abdominal pain	8y 7m	M	C	AD, Asthma	Y	N	restricting milk	9	Normal	No pathologic diagnosis	0
	**2**	OE	A	none	9y 4m	M	C			N	added milk		Normal	Focally inflamed squamous mucosa	15
**E22**	**1**	OE	A	obstructive	14y 6m	M	C	none	Y	N	open diet	5	Friable mucosa, furrows	Inflamed squamous mucosa	50
	**2**	OE	A	abdominal pain	15y 0m	M	C			N	removed milk and eggs		Furrows	Mildly inflamed squamous mucosa	15
**E23**	**1**	OE	A	obstructive, GER	11y 2m	M	C	AD, AR, Asthma	Y	N	restricting peanut, wheat, shellfish, milk (cow's), soy, egg, potato, tomato, corn	1	Edema, furrows	Increased intraepithelial lymphocytes and eosinophils with reactive epithelial cells	30
	**2**	E	I	none	11y 4m	M	C			N	restricting all foods and added amino acid based elemental formula		Furrows	Squamous mucosa with few intraepithelial eosinophils and mild to moderate epithelial changes	8

**E24**	**1**	OE	A	GER, abdominal pain	13y 1m	M	C	Asthma	Y	N	open diet	4	Normal	Inflamed squamous mucosa	20
	**2**	OE	A	abdominal pain	13y 5m	M	C			N	removed milk		Normal	Inflamed squamous mucosa with increased infiltrating lymphocytes and reactive epithelial changes	23
**E25**	**1**	O	A	none	4y 0m	M	C	Asthma	Y	N	removed egg, pork, turkey, chicken, apple, oat and corn	6	Furrows	Focally inflamed squamous mucosa	40
	**2**	O	I	none	4y 6m	M	C			N	removed egg, wheat, milk and corn		Furrows	Minimally inflamed squamous mucosa with very rare eosinophils	0
**E26**	**1**	O	I	GER, abdominal pain	11y 9m	M	AA	AD, AR, Asthma	Y	N	removed chicken, milk, nuts, peanuts, soy, strawberry, wheat, pork, lamb, turkey, peaches	Single time point	Normal	Mild esophagitis	8
**E27**	**1**	O	A	GER	2y 7m	M	C	AD	Y	N	open diet	4	Normal	Squamous mucosa with basal cell hyperplasia, elongation papillae, increased intraepithelial lymphocytes and eosinophils	22
	**2**	O	A	none	2y 11m	M	C			N	removed milk		White plaques	Moderate esophagitis with infiltrating lymphocytes, eosinophils and reactive epithelial changes.	20
**E28**	**1**	OE	A	none	9y 6m	M	C	AD, AR, Asthma	Y	N	removed peanuts and tree nuts	4	Furrows	Squamous mucosa with eosinophils and reactive epithelial changes	30
	**2**	O	I	unknown	9y 11m	M	C			N	removed milk, wheat, soy, egg and barley			Squamous mucosa with a few infiltrating lymphocytes and a rare single eosinophil compatible with very mild esophagitis	1
**E29**	**1**	OE	A	obstructive	10y 3m	M	C	none	Y	N	removed tree nuts	4	Friability, white plaques	Moderate to severe esophagitis with variable lymphocyte and eosinophil infiltrate and reactive epithelial changes	80
	**2**	O	I	obstructive	10y 7m	M	C			N	removed egg, milk and wheat		Furrows	Minimally inflamed squamous mucosa with an occasional eosinophil	0
**E30**	**1**	OE	A	obstructive	6y 3m	M	C	AD, Asthma	Y	N	removed soy and cantaloupe	2	Furrows	Variably inflamed squamous mucosa in association with reactive epithelial changes	60
	**2**	OE	A	abdominal pain	6y 6m	M	C			N	removed milk, egg, peanuts, tree nuts		Furrows	Esophagitis associated with reactive epithelial changes	40
**E31**	**1**	OE	A	none	10y 11m	F	C	AR, Asthma	Y	N	added milk and soy and restricting peanuts, tree nuts	5	Furrows	Variably inflamed squamous mucosa	50
	**2**	OE	A	obstructive	11y 4m	F	C			N	removed wheat		Furrows	Squamous mucosa with focally severe inflammation	40
**E32**	**1**	OE	A	none	10y 3m	M	C	none	Y	N	restricting milk and eggs	4	Normal	Squamous mucosa with some intraepithelial eosinophils	15
	**2**	OE	A	none	10y 7m	M	C			N	removed wheat		Normal	Squamous mucosa with many intraepithelial eosinophils and mild reactive epithelial changes	28

**E33**	**1**	OE	A	none	15y 11m	M	C	AR	Y	N	open diet	4	Furrows, rings	Esophagitis with numerous intraepithelial eosinophil granules, associated with reactive epithelial changes	15
	**2**	OE	I	none	16y 4m	M	C			N	removed milk		Rings	Minimally inflamed squamous mucosa	1
