# Supplementary figures and images for: Inflammation-associated microbiota in pediatric eosinophilic esophagitis
Source: Microbiome. 2015 Jun 1;3:23. doi: 10.1186/s40168-015-0085-6 (PMC4450515; doi:10.1186/s40168-015-0085-6)

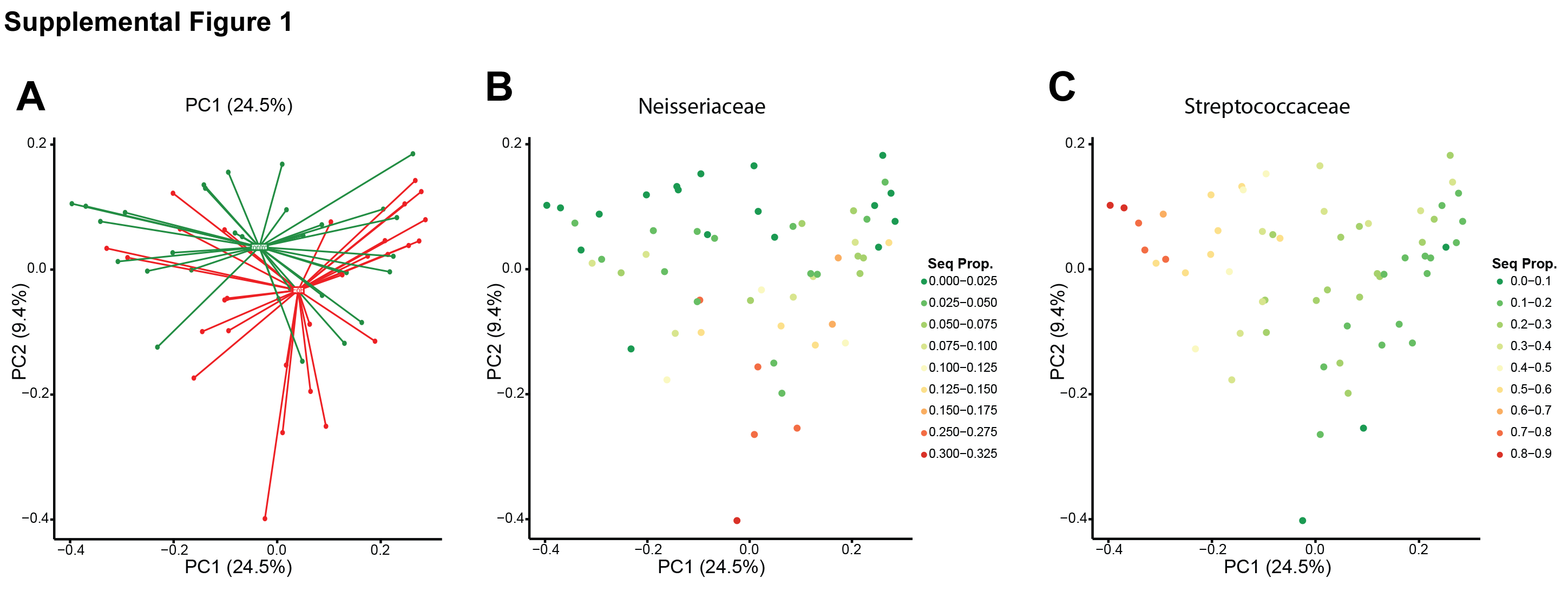

Supplement: Additional file 4: Figure S1. — Ordination comparing EoE and non-EoE control samples. (A) Pairwise distances were calculated between samples, then data plotted using Principal Coordinates Analysis. The centroid of the non-EoE control samples and the EoE samples are indicated on the plot. Lines connect each sample to the appropriate centroid. (B) and (C) show the same plot color-coded according the proportion of Neisseria and Streptococcus present in each sample. [file 40168_2015_85_MOESM4_ESM.tiff]

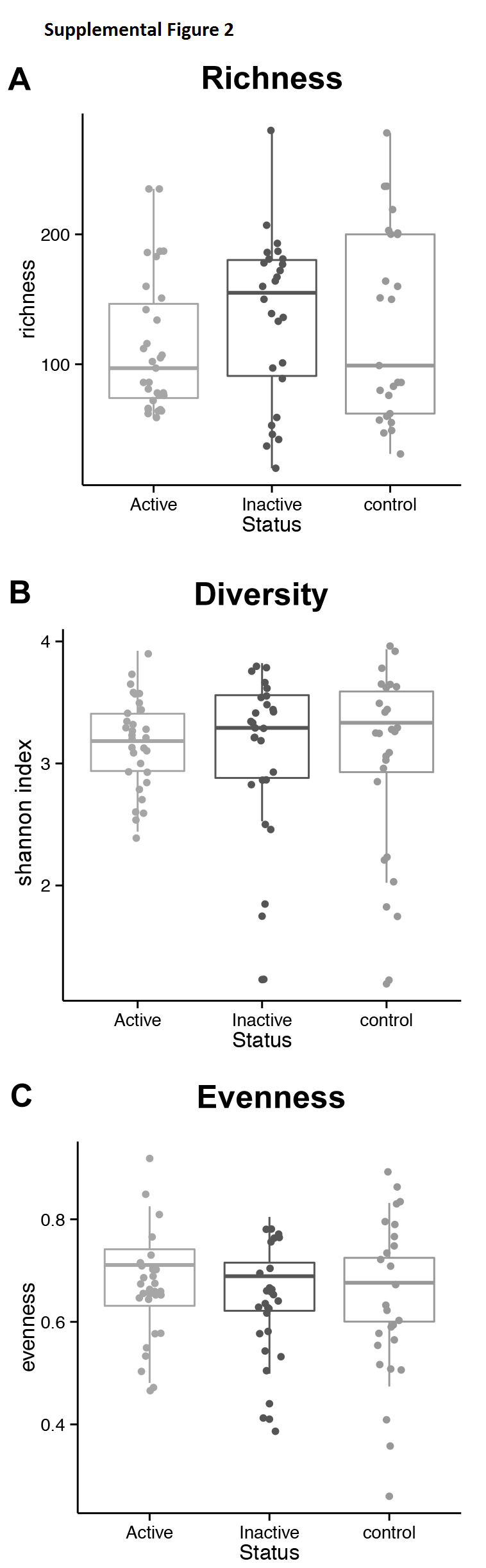

Supplement: Additional file 5: Figure S2. — Richness, Shannon diversity, and evenness indexes. Comparisons between control, inactive EoE, and active EoE using a Wilcoxon rank-sum test. [file 40168_2015_85_MOESM5_ESM.tif]

## Supplemental Figure 3

### Streptococcus

**A**

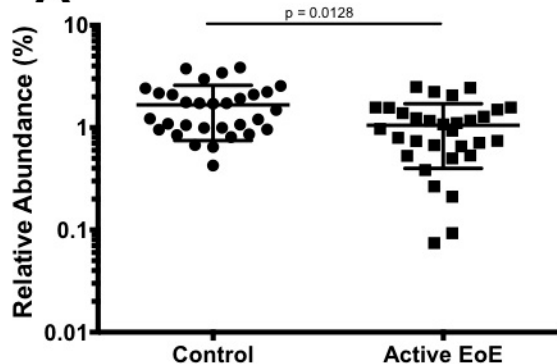

### Atopobium

**B**

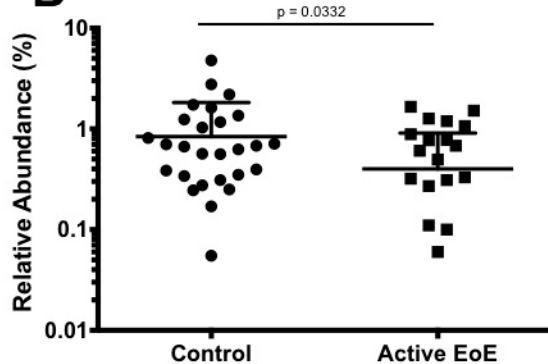

Supplement: Additional file 6: Figure S3. — Relative abundance for genus Streptococcus and Atopobium in active EoE versus non-EoE controls. [file 40168_2015_85_MOESM6_ESM.pdf]
